# A newborn screening approach to diagnose 3‐hydroxy‐3‐methylglutaryl‐CoA lyase deficiency

**DOI:** 10.1002/jmd2.12118

**Published:** 2020-04-14

**Authors:** Jan Václavík, Lucie Mádrová, Štěpán Kouřil, Julie de Sousa, Radana Brumarová, Hana Janečková, Jaroslava Jáčová, David Friedecký, Mária Knapková, Leo A. J. Kluijtmans, Sarah C. Grünert, Frédéric M. Vaz, Nils Janzen, Ronald J. A. Wanders, Ron A. Wevers, Tomáš Adam

**Affiliations:** ^1^ Institute of Molecular and Translational Medicine, Faculty of Medicine and Dentistry, Palacký University Olomouc Olomouc Czech Republic; ^2^ Laboratory of Inherited Metabolic Disorders, Department of Clinical Chemistry University Hospital in Olomouc Olomouc Czech Republic; ^3^ Department of Mathematical Analysis and Applications of Mathematics, Faculty of Science Palacký University Olomouc Olomouc Czech Republic; ^4^ Banská Bystrica Children's University Hospital Banská Bystrica Slovakia; ^5^ Translational Metabolic Laboratory, Department of Laboratory Medicine Radboud University Medical Centre GA Nijmegen Netherlands; ^6^ Department of General Pediatrics, Adolescent Medicine and Neonatology Medical Center – University of Freiburg, Faculty of Medicine Freiburg Germany; ^7^ Laboratory Genetic Metabolic Diseases, Department of Clinical Chemistry Amsterdam Netherlands; ^8^ Screening‐Labor Hannover Hannover Germany; ^9^ Department of Clinical Chemistry Hannover Medical School Hannover Germany

**Keywords:** 3‐hydroxy‐3‐methylglutaryl‐coenzyme A lyase deficiency, acylcarnitines, biomarkers, HMG‐CoA lyase, metabolomics, newborn screening, organic acids

## Abstract

3‐Hydroxy‐3‐methylglutaryl‐coenzyme A lyase deficiency (HMGCLD) is a rare autosomal recessively inherited metabolic disorder. Patients suffer from avoidable neurologically devastating metabolic decompensations and thus would benefit from newborn screening (NBS). The diagnosis is currently made by measuring dry blood spot acylcarnitines (C5OH and C6DC) followed by urinary organic acid profiling for the differential diagnosis from several other disorders. Using untargeted metabolomics (reversed‐phase UHPLC coupled to an Orbitrap Elite hybrid mass spectrometer) of plasma samples from 5 HMGCLD patients and 19 age‐matched controls, we found 3‐methylglutaconic acid and 3‐hydroxy‐3‐methylglutaric acid, together with 3‐hydroxyisovalerylcarnitine as the most discriminating metabolites between the groups. In order to evaluate the NBS potential of these metabolites we quantified the most discriminating metabolites from untargeted metabolomics in 23 blood spots from 4 HMGCLD patients and 55 controls by UHPLC tandem mass spectrometry. The results provide a tool for expanded NBS of HMGCLD using tandem mass spectrometry. Selected reaction monitoring transition 262/85 could be used in a first‐tier NBS analysis to screen for elevated 3‐hydroxyisovalerylcarnitine. In a positive case, a second‐tier analysis of 3‐hydroxy‐3‐methylglutaric acid and 3‐methylglutaconic acid in a dry blood spot using UHPLC tandem mass spectrometry instruments confirms the diagnosis. In conclusion, we describe the identification of new diagnostic biomarkers for HMGCLD and their application in NBS in dry blood spots. By using second‐tier testing, all patients with HMGCLD were unequivocally and correctly diagnosed.

**Abbreviations**
3H3MG‐A: 3‐hydroxy‐3‐methylglutaric acid
3H3MG‐C: 3‐hydroxy‐3‐methylglutarylcarnitine
3HIV‐A: 3‐hydroxyisovaleric acid
3HIV‐C: 3‐hydroxyisovalerylcarnitine
3MC‐C: 3‐methylcrotonylcarnitine
3MG‐A: 3‐methylglutaric acid
3MG‐C: 3‐methylglutarylcarnitine
3MGC‐A: 3‐methylglutaconic acid
3MGC‐C: 3‐methylglutaconylcarnitine
C5OH: acylcarnitine with acyl consisting of 5 carbon atoms and hydroxy group
C6DC: acylcarnitine with acyl consisting of 6 carbon atoms and carboxyl group
CID: collision induced dissociation
DBS: dried blood spot
IV‐C: isovalerylcarnitine
MCCD: 3‐methylcrotonyl‐CoA carboxylase deficiency
MGCA: 3‐methylglutaconic aciduria
MRM: multiple reaction monitoring
MSI: Metabolomic Standard Initiative
NBS: newborn screening
OPLS‐DA: orthogonal partial least squares discriminant analysis
HDI: highest density interval
HMGCLD: 3‐hydroxy‐3‐methylglutaryl‐coenzyme A lyase deficiency
RT: retention time
SRM: selected reaction monitoring
VIP: importance in projection

**SYNOPSIS**

In this article we describe new blood biomarkers of 3‐hydroxy‐3‐methylglutaryl‐CoA lyase deficiency that allow newborn screening from initial dried blood spots without the necessity of additional sampling.

## 1 INTRODUCTION

3‐Hydroxy‐3‐methylglutaryl‐coenzyme A lyase deficiency (HMGCLD, OMIM 246450) is a rare autosomal recessively inherited metabolic disorder caused by mutations in the *HMGCL* gene. The mitochondrial enzyme is responsible for catalyzing the cleavage of HMG‐CoA to acetyl‐CoA and acetoacetic acid. This conversion is a common last step in leucine catabolism and ketogenesis from fatty acids. Patients with HMGCLD present with a diagnostic urinary pattern of elevated organic acids such as 3‐hydroxyisovaleric acid (3HIV‐A), 3‐methylglutaconic acid (3MGC‐A), 3‐hydroxy‐3‐methylglutaric acid (3H3MG‐A), 3‐methylglutaric acid (3MG‐A) and in some cases 3‐methylcrotonylglycine. Plasma of these patients contains elevated levels of 3‐hydroxyisovalerylcarnitine (3HIV‐C),^1^ 3‐methylglutarylcarnitine (3MG‐C),^2^ and three isomers of 3‐methylglutaconylcarnitine (3MGC‐C).^3^ Patients may suffer from severe attacks of metabolic decompensation with lethargy, seizures, hypotonia, vomiting and acidosis with hypoketotic hypoglycemia that may result in irreversible neurological damage.^4^ Most patients manifest within the first year of life. Patients diagnosed at an early stage with careful dietary management may avoid metabolic crises and could have good prognosis.

For diagnostics, tandem mass spectrometry based selective metabolic screening methods on dried blood spot (DBS) samples are commonly used with a joint selected reaction monitoring (SRM) transition for C4‐DC and C5‐OH acylcarnitines. However, the SRM transition 262/85 used to analyze these acylcarnitines represents four isobaric acylcarnitines (3HIV‐C, 2‐methyl‐3‐hydroxybutyrylcarnitine, methylmalonylcarnitine, and succinylcarnitine), pointing to many different inborn errors of metabolism (3‐methylcrotonyl‐CoA carboxylase deficiency [MCCD], HMGCLD, β‐ketothiolase deficiency, 3‐methylglutaconic aciduria [MGCA], 2‐methyl‐3‐hydroxybutyryl‐CoA dehydrogenase deficiency, methylmalonic acidemia, multiple carboxylase deficiency, succinyl‐CoA ligase deficiency and mutation in genes encoding the α‐subunit and the β‐subunit of the ADP‐forming succinyl‐CoA synthetase).^5^ In order to distinguish between these disorders, a patient must be recalled for urine sampling and/or analysis of enzyme activities. MCCD has the highest incidence (1:40 000) among the disorders listed above. However, it is estimated that about 95% of MCCD cases are benign^6^ and require no medical interventions.

In this paper we describe the detection of plasma acylcarnitines and organic acids related to the leucine degradation pathway in patients suffering from HMGCLD using untargeted metabolomics. 3‐Hydroxy‐3‐methylglutarylcarnitine (3H3MG‐C), previously considered undetectable in plasma of HMGCLD patients,^7^ was found for the first time. Furthermore, organic acid counterparts of elevated acylcarnitines were detected in plasma of HMGCLD patients as the most discriminating metabolites. Determination of these metabolites could be implemented in newborn screening (NBS) programs in order to detect patients suffering from HMGCLD. A following experiment of targeted analysis of the most discriminating metabolites between patient and control groups using DBS samples showed applicability of these metabolites in second‐tier LC‐MS/MS method within NBS programs to specifically diagnose HMGCLD, which will facilitate early recognition, appropriate treatment and prevention of metabolic decompensations in this disease.

## 2 MATERIALS AND METHODS

### 2.1 Chemicals

Methanol, ethanol, water, and formic acid (LC‐MS quality) were purchased from Sigma‐Aldrich (St. Louis, Missouri). The following chemical standards were used for feature identification/quantification: isovalerylcarnitine (IV‐C), 3‐methylcrotonylcarnitine (3MC‐C), and 2‐methylbutyrylcarnitine were purchased from Sigma‐Aldrich (Switzerland). Adipoylcarnitine and tiglylcarnitine from Sigma‐Aldrich (Austria). 3MG‐C from Avanti (Massachusetts). 3MGC‐A and 3H3MG‐A from Sigma‐Aldrich (Germany). Adipate from Fluka (Germany) and 3MG‐A from Sigma‐Aldrich (India).

### 2.2 Samples

The study was conducted in accordance with the Declaration of Helsinki and adhered to Good Clinical Practice guidelines. Approval for the protocol was obtained from the joint ethics committee of the Medical Faculty of Palacký University and University Hospital Olomouc. Plasma samples for untargeted metabolomics were obtained from five HMGCLD patients—three girls (age 4, 17 days and 8 years) and two boys (1 and 5 years)—whose diagnosis was confirmed by enzyme and genetic testing. Control plasma samples were obtained from 19 children (9 boys and 10 girls between 2 and 17 years of age) into 5 mL vacuum sampling tube K_3_EDTA Vacuette, Greiner (Germany) at room temperature and centrifuged for 10 minutes at 3000*g*. Separated plasma was stored at −80°C until analysis. Shipping of samples between collaborating laboratories was conducted via 48 hours‐courier on sufficient dry ice.

For targeted LC‐MS/MS analysis, three types of control DBS samples were used: healthy newborns (ConA, n = 14) sampled a maximum of 3 weeks before analysis (representing controls for comparison with newborns suffering from HMGCLD), healthy newborns (ConB, n = 14) sampled approx. Six years before analysis (representing controls for comparison with the aged patient DBS samples we managed to acquire for this study) and disease‐free individuals of age between 1 month and 15 years old (ConC, n = 27) sampled a maximum of 2 months before analysis (representing controls matching the age of HMGCLD patients that we managed to acquire for this study). All control samples were collected in the Laboratory for Inherited Metabolic Disorders (Department of Clinical Biochemistry, University Hospital Olomouc, Czech Republic).

DBS samples of genetically confirmed HMGCLD patients, were obtained anonymized from co‐working laboratories from Freiburg (Germany) (Pt1—a treated girl with low protein diet and l‐carnitine supplementation, 20 DBS sampled at age 4.4 to 15.2 years) and Banská Bystrica (Slovakia) (Pt4—a newborn sample from a boy diagnosed at 10th day of age treated since with low protein diet and l‐carnitine supplementation). Two more DBS samples of suspected HMGCLD patients (Pt2 and Pt3—two girls, 5.6 and 10.0 years old) from Hannover (Germany) were included in the study, even though their diagnoses were not confirmed by genetic testing yet.

### 2.3 Methods

Plasma samples of five HMGCLD patients were analyzed together with a group of controls using untargeted metabolomics via liquid chromatography coupled with high‐resolution mass spectrometry (LC‐HRMS) to define discriminating compounds. Detailed information about performed untargeted metabolomics and data processing is enclosed in Supporting information S1. After data processing and statistical evaluation, physiological concentrations of the most elevated metabolites were determined using 55 control DBS samples using LC‐MS/MS analysis. Also, pathological concentrations of these metabolites were calculated in DBS samples of HMGCLD patients. An overview of performed experiments is depicted in Supporting information S2. All data are reported in accordance to Metabolomic Standard Initiative (MSI) reporting requirements.^8^

#### 2.3.1 Identification of discriminating metabolites

Following MSI principles for metabolite identification, the identity of acylcarnitines and organic acids related to leucine degradation pathway intermediates was determined by comparing the accurate mass of precursor ions, fragmentation spectra and RT's with commercially available standards (MSI level 1). In absence of commercial standards, discriminating metabolites were putatively annotated based on accurate masses and comparison of fragmentation spectra and a region of elution from chromatographic column with a similar compound class metabolite for which a commercial standard was available (MSI level 2). Not all discriminating metabolites could be annotated and those were marked as unknowns (MSI level 4).

#### 2.3.2 Targeted LC‐MS/MS analysis of DBS

In order to evaluate the diagnostic potential of discriminating metabolites from untargeted metabolomic analysis for NBS, 23 DBS samples of 4 HMGCLD patients (one patient provided 20 samples over the course of 11 years of life) were analyzed together with 60 controls by LC system Sciex Exion AD which was coupled to a mass spectrometer (QTrap 6500+; Sciex, Framingham, Massachusetts) in scheduled multiple reaction monitoring (MRM) mode. The targeted LC‐MS/MS method consisted of MRM transitions representing metabolites found by untargeted metabolomic experiment and MRM transitions for metabolites that could be theoretically expected (acylglycines, acylcarnitines and organic acids related to the affected pathway). Targeted LC‐MS/MS method was modified based on original work from Körver‐Keularts et al^9^ and is described in Supporting information S3.

Sample preparation involved dissection of one disk (3.0 mm) from each DBS sample and extracted in methanol (100 μL) containing isotopically labeled internal standards; isovalerylcarnitine‐D9 and methylmalonate‐D3. After 20 minutes shaking at 405 RPM, extracts were lyophilized, reconstituted in 0.1% formic acid (100 μL), centrifuged at 21 300*g* for 10 minutes at 4°C and 90 μL of supernatants were transferred into UHPLC vials. A six‐point calibration mixture of three standards (3HIV‐A, 3MGC‐A and 3H3MG‐A) was used to calculate their concentrations in blood using a dilution factor of 62.5 as a 3.0 mm DBS punch contains 1.6 μL of blood^10^ which was extracted with 100 μL of solvent.

## 3 RESULTS

### 3.1 Untargeted metabolomic analysis

Plasma samples of five HMGCLD patients were analyzed together with controls via untargeted metabolomic analysis. After data processing and filtering, a total of 429 unique *m*/z features were found. PCA as unsupervised statistical method with cumulative variance explained by the first two principal components of 62.3% showed clear separation of clusters pointing to distinct metabolite differences between patient and control groups (see Supporting information S4).

Supervised statistical methods such as Bayesian volcano plot (see Supporting information S5) with colored HDI distance levels and VIP plot from OPLS‐DA were used to determine the most elevated metabolites in a group of patients, see Table 1. The list of discriminating metabolites contains all known plasma biomarkers of the disease (3HIV‐C, 3MG‐C, 3MGC‐C). Furthermore, it also comprises two free organic acids (3MGC‐A and 3H3MG‐A) derived from accumulated acyl‐CoAs prior to the metabolic block that are known biomarkers of the disease in the urine, although they were never reported as being elevated in blood samples from affected patients. Also, 3HIV‐A is listed in Supporting information S6 alongside 3MGC‐A and 3H3MG‐A as it is the organic acid counterpart to a known plasma biomarker of HMGCLD (3HIV‐C).

**TABLE 1:** Most elevated metabolites according to VIP scores from OPLS‐DA in untargeted metabolomic analysis of plasma samples of HMGCLD patients

Metabolite	[M+H]^+^	RT (min)	VIP score	HDI distance	Fold‐change^a^	MSI identification
3MG‐C	290.1598	4.38	3.04	5.1	63.2	Level 1
3MGC‐A	127.0383^b^	5.49	2.68	3.4	151.4	Level 1
3HIV‐C	262.1649	3.65	2.47	2.9	137.1	Level 2
3H3MG‐A	163.0601	3.00	2.47	2.0	359.3	Level 1
3MGC‐C	288.1441	4.42	2.24	2.4	50.5	Level 2
Unknown	247.0391	5.48	2.18	2.2	55.2	Level 4
Unknown	321.0854	3.97	2.03	0.6	135.8	Level 4

a Calculated as a mean of patient´s peak area divided by mean of ConC peak area.

b In‐source fragment with larger peak area than molecular ion.

Apart from known plasma biomarkers of HMGCLD patients (3HIV‐C, 3MG‐C, and 3MGC‐C) other acylcarnitine species derived from intermediates in the leucine degradation pathway turned out to be elevated when compared to controls (see Supporting information S6). Especially, the identification of 3H3MG‐C deserves attention as its CoA analogue is an intermediate of the leucine degradation pathway which is, as of yet, known as the single substrate for the HMGCL enzyme and was previously considered undetectable.^7^

### 3.2 Targeted LC‐MS/MS analysis

**FIGURE 1:**
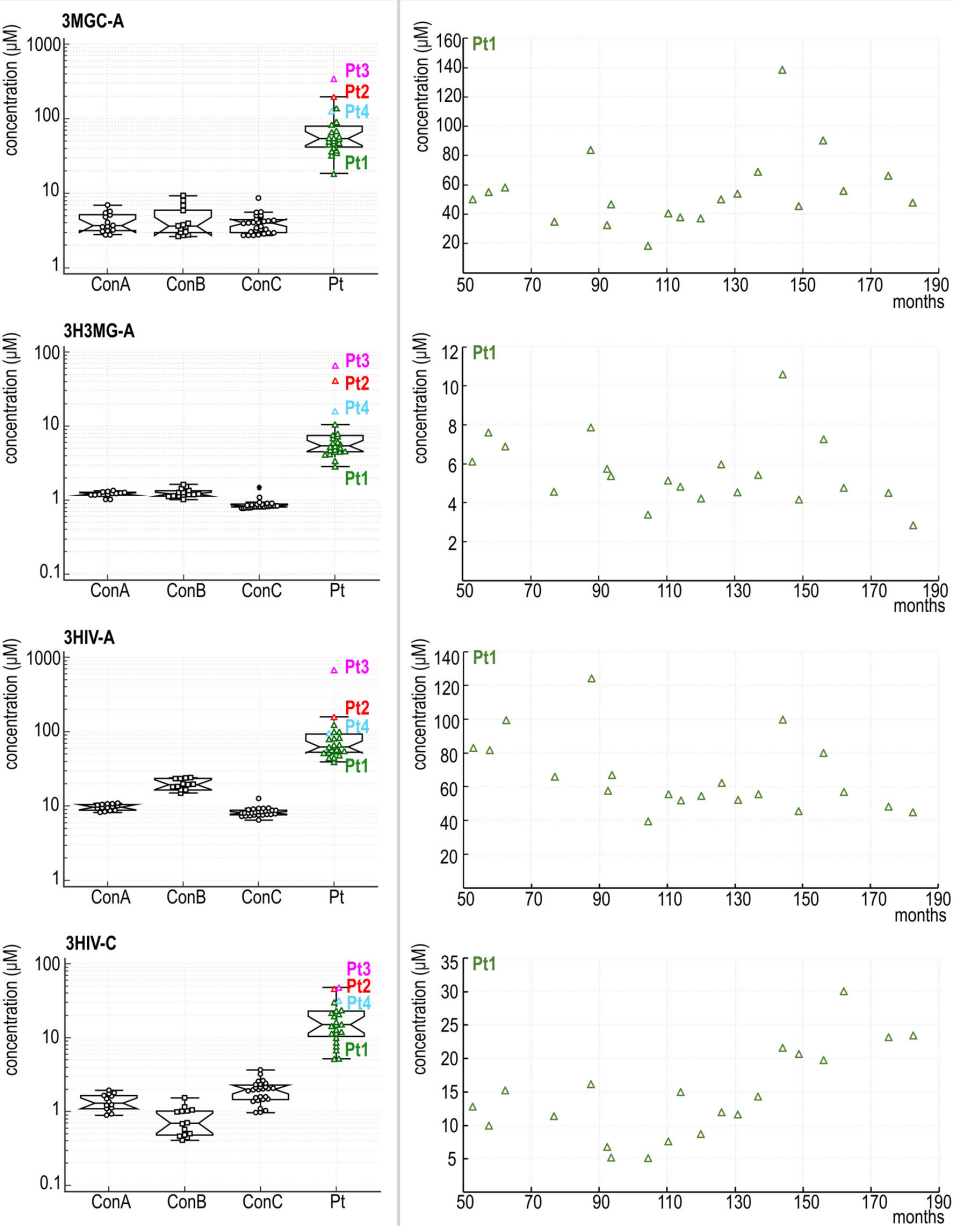
A, Boxplots of the 3MGC‐A, 3H3MG‐A, 3HIV‐A, and 3HIV‐C concentration of patients (Pt) and controls (ConA, ConB, and ConC) from targeted LC‐MS/MS analysis of dried blood spot samples. A logarithmic scale was used for the y‐axis, allowing better comparability between the different groups of controls. B, The concentration of the relevant metabolites in blood of patient 1 was followed over time between ages 4.4 and 15.2 years

The concentration of metabolites in blood calculated (described in Section 2.3.2) from DBS samples of HMGCLD patients and controls is provided in Table 2. Box plots show a clear separation between the healthy population and HMGCLD patients without any overlap (ie, statistically significant difference between the respective groups) for 3MGC‐A, 3H3MG‐A, 3HIV‐A, and 3HIV‐C, see Figure 1.

**TABLE 2:** The metabolite concentrations in blood of controls and HMGCLD patients with calculated fold‐changes according to targeted analysis of dried blood spot samples

Metabolite (μM)	ConA	ConB	ConC	Mean Pt1	Pt2	Pt3	Pt4	Fold‐change1^a^	Fold‐change2^b^
3MGC‐A	4.1 ± 2.5	4.6 ± 4.2	4.2 ± 3.7	55.5 ± 51.7	195.9	343.2	126.9	13.3	53.2
3H3MG‐A	1.2 ± 0.2	1.3 ± 0.3	0.9 ± 0.4	5.6 ± 3.6	40.0	64.9	16.1	6.3	45.2
3HIV‐A	9.5 ± 2.1	19.3 ± 6.2	8.6 ± 3.6	66.2 ± 43.9	158.5	659.8	95.8	7.7	35.4
3HIV‐C	1.3 ± 1.0	0.8 ± 0.7	1.9 ± 1.5	14.6 ± 13.6	45.7	47.5	31.7	7.6	21.8

a Calculated as a mean of patient 1 (treated patient) concentrations divided by mean of ConC concentration.

b Calculated as a mean of patient 2, 3 and 4 (untreated at a time of sampling) concentrations divided by mean of ConC concentration.

## 4 DISCUSSION

Patients suffering from HMGCLD may exhibit abnormal plasma levels of ammonia, lactate, 3MG‐C,^2^ 3HIV‐C,^1^ and three isomers of 3MGC‐C.^3^ None of these metabolites is a selective biomarker for the disease making the differential diagnosis of HMGCLD directly from DBS impossible. The diagnosis of HMGCLD relies on urinary organic acid profile characterized by elevated concentrations of 3HIV‐A, 3H3MG‐A, 3MGC‐A, 3MG‐A, adipic acid, and in some cases 3‐methylcrotonylglycine.^11^ Urine sampling of these patients usually occurs during a (first) metabolic decompensation with manifestation of symptoms, such as recurrent vomiting, seizures, and impaired vigilance. Common laboratory findings of the metabolic crisis include hypoglycemia, acidosis, hyperammonemia, an increased anion gap, and elevated transaminase activities. The majority of HMGCLD patients (92%) become symptomatic within the first year of life and about half of them within the neonatal period. Over 70% of patients exhibit brain MRI abnormalities^4^ presumably due to metabolic crises that occur early in their lives. Recognition of the HMGCLD diagnosis at (very) early age may lead to early start of appropriate clinical management, and the number of future metabolic decompensations may, therefore, be limited. Most HMGCLD patients show a favorable outcome with normal psychomotor development, which demonstrates the need for early recognition and diagnosing the disease. As in other organic acidurias, a timely diagnosis is crucial for overall quality of life of HMGCLD patients and demonstrates that this disease is a perfect candidate for NBS programs. The diagnosis is usually confirmed by enzyme and/or genetic testing.

To the best of our knowledge, there is no reliable source of information about worldwide incidence of HMGCLD, nevertheless, it seems to be extremely rare. There are only few studies reporting higher frequencies in certain populations such as in Saudi Arabia, Brazil, Portugal and Spain, however, all less than 1/100 000 live births.^11^, ^12^, ^13^ According to the Uniform Screening Panel,^14^ HMGCLD scored 16th from the top of 84 inherited metabolic disorders reconsidered for implementation into NBS programs in the United States. The low incidence of the disease may limit scientific progress in the development of specific analytical methods useful for timely diagnosis of HMGCLD without the need for confirmation analysis on urine samples.

Using untargeted metabolomic analysis of HMGCLD patient plasma, 3MGC‐A and 3H3MG‐A were found among the most discriminating metabolites between patient and control group. Subsequent LC‐MS/MS analysis of DBS samples from HMGCLD patients and controls was used to determine the physiological concentration range of 3MGC‐A (4.1 ± 2.5 μM), 3H3MG‐A (1.2 ± 0.2 μM), 3HIV‐A (9.5 ± 2.1 μM), and 3HIV‐C (1.3 ± 1.0 μM) in blood spots of newborns and show significantly increased pathological levels of these metabolites in HMGCLD patients. It is worth noticing that elevations of proposed diagnostic metabolites in newborn patient 4 (although milder than in the other three symptomatic patients) are approximately one order of magnitude higher than the highest value in controls.

In conclusion, our results provide a tool for expanded NBS of HMGCLD using tandem mass spectrometry. The SRM transition 262/85 could be used in a first‐tier NBS analysis to screen for elevated 3HIV‐C. In a positive case, a second‐tier analysis of 3H3MG‐A and 3MGC‐A using an LC‐MS/MS instrument may confirm the diagnosis. Given the rarity of the diseases a larger study is warranted to prove the practicality of this approach and to develop an optimal screening algorithm for HMGCLD. The methodology used in the article could be repeated for others disorders characterized by the elevated C5OH, leading to more diagnostically precise second tier tests that would facilitate timely diagnosis in the blood spot. Authors are looking for collaboration in this respect.

## CONFLICT OF INTEREST

The authors declare no conflicts of interest.

## ETHICS STATEMENT

All procedures followed were in accordance with the ethical standards of the responsible committee on human experimentation (institutional and national) and with the Helsinki Declaration of 1975, as revised in 2000. All patient and control samples were pseudonymized in the study. This article does not contain any studies with animal subjects performed by any of the authors.

## Supporting information


**Data S1.** Supporting information.

**Data S2.** An overview of performed experiments and data analysis.

**Data S3.** LC‐MS/MS analysis of DBS.

**Data S4.** Two‐dimensional score plot of unsupervised PCA analysis of HMGCLD patient plasma (Pt, blue) and controls (Con, pink). The tight green cluster of QC samples show a good stability of the analysis. Circled areas represent 75% confidence ellipses.

**Data S5.** Bayesian volcano plot with colored HDI distance levels for the untargeted analysis. The dark red points stand for the non‐significant compounds. The potential biomarkers are depicted in blue‐green colors, located in the upper right corner for patients and in the upper left corner for controls, respectively. *No metabolites detected in these HDI distance levels.

**Data S6.** Acylcarnitine profile of five HMGCLD patients compared to 19 healthy controls in plasma. Elevated organic acids found in plasma samples of HMGCLD patients compared to 19 healthy controls.
