# Supplementary material for: A newborn screening approach to diagnose 3‐hydroxy‐3‐methylglutaryl‐CoA lyase deficiency
Source: JIMD Rep. 2020 Apr 14;54(1):79–86. doi: 10.1002/jmd2.12118 (PMC7358667; doi:10.1002/jmd2.12118)
Supplement: Supplementary file 1 — Data S1. Supporting information. [file JMD2-54-79-s001.docx]

# A newborn screening approach to diagnose 3-hydroxy-3-methylglutaryl CoA lyase deficiency

# Supplement materials S1

### **Untargeted metabolomics**

Plasma samples were thawed at 4 °C and a 50 µL heparinized plasma aliquot was transferred into a polypropylene microcentrifuge tube. Ice-cold methanol/ethanol (1:1, 200 µL) was added to each aliquot for protein precipitation. The samples were mixed for 30 s, incubated at -80 °C overnight and then centrifuged at 21 300 x *g* for 15 min at 4 °C. The supernatant (220 µL) was freeze-dried and reconstituted in water with formic acid (0.1 %, 100 µL), vortexed for 15 s and centrifuged at 21 300 x *g* for 15 min at 4 °C. System QC samples were prepared from a pool of all patient samples and a pool of all control samples in a ratio of 1:1 in order to avoid a dilution effect of a significantly larger number of control over patient samples. All samples were analyzed in one batch consisting of conditioning QC samples (7), blank samples (one at the beginning and one at the end of the batch), QC samples for which data dependent MS^2^ spectra were acquired (8), a mix of standards to distinguish between isobaric compounds (4) and system QC samples (every 6^th^ injection). Plasma samples from patient and control groups were analyzed in randomized fashion.

All samples in the batch were applied (5 μL) to a silica-based bonded reversed-phase Acquity HSS T3 column (Waters, Milford, MA, USA, 100 × 2.1 mm; 100 Å, 1.8 μm) maintained at 40 °C within a column compartment of an Ultimate 3000 RS LC system (Dionex, Sunnyvale, CA, USA). Samples were injected using a sampling loop of 40 μL with wash cycles of mobile phase B between injections. The chromatographic system was coupled to an Orbitrap Elite hybrid mass spectrometer (Thermo Fisher Scientific, MA, USA). The mobile phase A consisted of 0.1 % formic acid in water (pH 2.6) and mobile phase B consisted of 0.1 % formic acid in methanol:water solution (99:1). The temperature of the autosampler was held constant at 4 °C. The analyses were run at a flow rate of 400 µL/min at pressure ranging 410 – 710 bar throughout the run. The gradient program was as follows: t = 0.0 – 1.0 min, 1 % B; t = 16.0 – 20.0 min, 100 % B; t = 21.0 – 25.0 min, 1 % B. Samples were analyzed in positive (+ 2.8 kV) mode using electrospray ionization. The temperatures of the ion source and transfer tube were both set to 350 °C. Sheath-, auxiliary- and sweeping gases were set to 50, 16 and 0 arbitrary units, respectively. Full scan mode analyses were performed at resolution of 120 000 full width at half maximum within *m/z* range of 90 – 1000. A mass accuracy below 2 ppm was achieved by employing external mass calibration (Positive calibration mix, Thermo Scientific, San Jose, USA) before analyzing the batch and online mass calibration using internal lock mass with diisooctyl phthalate (ion *m/z* 391.2843).

For data-dependent fragmentation methods, a reject mass list was generated from all *m/z* features found in blank samples of an intensity above 30 000 counts. Data dependent MS^2^ experiments were performed at resolution 15 000 full width at half maximum via two different fragmentation techniques applied on QC samples. In every scan cycle, the top five most intensive ions within four *m/*z ranges 90 – 200, 200 – 400, 400 – 600 and 600 – 1000 (an injection of QC sample per each *m/z* range) underwent collision induced dissociation (CID) with isolation width of 1 Da. Energy used during CID fragmentation was 35 units of normalized collision energy. High-energy collisional dissociation data dependent MS^2^ spectra were acquired applying the same settings as in the case of CID spectra except for used collision energy - 50 units of normalized collision energy.

#### **Data processing**

Peak-picking was conducted with Compound Discoverer 3.0 (Thermo Fisher Scientific, MA, USA) including peak area integration, gap-filling, retention time (RT) alignment and removal of peaks below 10 000 of intensity. Only peaks with RT below 12 min were accounted for since a majority of small molecule metabolites elute before 12^th^ min of the analysis A complete list of Compound Discoverer parameters set for peak-picking is listed below. Merging of adducts, isotopes and in-source fragments was conducted in R software (v3.5.0) as described in Kouřil et al^1^. Locally estimated smoothing signal correction was applied^2^ and metabolites/features with a coefficient of variation of QC samples higher than 30% were excluded from further data curation using the R package Metabol.^3^ The data were transformed by probabilistic quotient normalization^4^ and natural logarithm was applied for their scaling^5^ before subsequent statistical analysis. Outliers were detected groupwise by the ROBPCA method combining robust covariance estimation with projection pursuit techniques^6^ implemented in the package Metabol. This resulted in a reduction of the number of control samples from 21 to 19.

Multidimensional statistical methods such as principal component analysis (PCA) and orthogonal partial least squares discriminant analysis (OPLS-DA) were applied with the use of Metabol for statistical evaluation of acquired data from untargeted metabolomic analysis. A Bayesian volcano plot with coloured highest density interval (HDI) distance levels^7^ and variable importance in projection (VIP) plot from OPLS-DA were employed to determine the most discriminating metabolites between patients and controls. Only metabolites with the distance of HDI boundary from zero ≥ 2 and metabolites with VIP scores above 2 were considered significant and if possible, structurally identified.

**Complete list of Compound Discoverer parameters setting for peak-picking**

Search name: 2019_03_08_hmgcld

Search description: -

Search date: 3/8/2019 9:43:01 AM

Created with Discoverer version: 3.0.0.294

[Input Files (0)]

-->Select Spectra (1)

[Select Spectra (1)]

-->Align Retention Times (2)

[Align Retention Times (2)]

-->Detect Compounds (3)

[Detect Compounds (3)]

-->Group Compounds (4)

[Group Compounds (4)]

-->Fill Gaps (16)

-->Search ChemSpider (8)

-->Assign Compound Annotations (9)

-->Predict Compositions (12)

-->Map to KEGG Pathways (23)

-->Map to Metabolika Pathways (25)

-->Search mzCloud (21)

-->Compound Class Scoring (26)

[Fill Gaps (16)]

-->Normalize Areas (19)

[Normalize Areas (19)]

-->Mark Background Compounds (15)

[Search ChemSpider (8)]

-->Apply mzLogic (22)

[Map to Metabolika Pathways (25)]

-->Apply mzLogic (22)

[Mark Background Compounds (15)]

[Apply mzLogic (22)]

[Assign Compound Annotations (9)]

[Predict Compositions (12)]

[Map to KEGG Pathways (23)]

[Search mzCloud (21)]

[Compound Class Scoring (26)]

[Differential Analysis (10)]

[Descriptive Statistics (18)]

------------------------------------------------------------------

Processing node 0: Input Files

------------------------------------------------------------------

Input Data:

- File Name(s) (Hidden):

X:\HMGCLD_project\6_Blank1.raw

X:\HMGCLD_project\7_QC6_CID1.raw

X:\HMGCLD_project\8_QC7_CID2.raw

X:\HMGCLD_project\9_QC8_CID3.raw

X:\HMGCLD_project\10_QC9_CID4.raw

X:\HMGCLD_project\11_QC10_HCD1.raw

X:\HMGCLD_project\12_QC11_HCD2.raw

X:\HMGCLD_project\22_QC14.raw

X:\HMGCLD_project\23_QC15.raw

X:\HMGCLD_project\26_ConC19_boy_13year.raw

X:\HMGCLD_project\28_ConC9_girl_19year.raw

X:\HMGCLD_project\30_QC16.raw

X:\HMGCLD_project\33_Pac4_boy_1year.raw

X:\HMGCLD_project\35_ConC4_boy_14year.raw

X:\HMGCLD_project\37_QC17.raw

X:\HMGCLD_project\39_ConC14_girl_16year.raw

X:\HMGCLD_project\43_ConC2_boy_17year.raw

X:\HMGCLD_project\44_QC18.raw

X:\HMGCLD_project\45_ConC16_girl_5year.raw

X:\HMGCLD_project\51_QC19.raw

X:\HMGCLD_project\53_Pac1_girl_8year.raw

X:\HMGCLD_project\54_ConC10_boy_8year.raw

X:\HMGCLD_project\58_QC20.raw

X:\HMGCLD_project\59_ConC5_girl_11year.raw

X:\HMGCLD_project\62_ConC13_girl_15year.raw

X:\HMGCLD_project\63_Pac2_girl_4day.raw

X:\HMGCLD_project\65_QC21.raw

X:\HMGCLD_project\71_ConC22_boy_13year.raw

X:\HMGCLD_project\72_QC22.raw

X:\HMGCLD_project\74_ConC8_boy_7year.raw

X:\HMGCLD_project\76_Pac5_girl_17day.raw

X:\HMGCLD_project\77_ConC6_boy_1year.raw

X:\HMGCLD_project\79_QC23.raw

X:\HMGCLD_project\81_ConC15_boy_11year.raw

X:\HMGCLD_project\84_ConC1_girl_2year.raw

X:\HMGCLD_project\86_QC24.raw

X:\HMGCLD_project\88_ConC12_boy_15year.raw

X:\HMGCLD_project\91_Pac3_boy_5year.raw

X:\HMGCLD_project\92_ConC3_girl_15year.raw

X:\HMGCLD_project\93_QC25.raw

X:\HMGCLD_project\96_ConC17_girl_4year.raw

X:\HMGCLD_project\99_ConC20_girl_4year.raw

X:\HMGCLD_project\100_QC26.raw

X:\HMGCLD_project\113_Blank4.raw

X:\HMGCLD_project\102_ConC18_boy_12year.raw

X:\HMGCLD_project\104_ConC21_girl_16year.raw

X:\HMGCLD_project\106_ConC7_boy_12year.raw

X:\HMGCLD_project\107_QC27.raw

X:\HMGCLD_project\110_QC28.raw

X:\HMGCLD_project\111_QC29.raw

X:\HMGCLD_project\112_Blank2.raw

X:\HMGCLD_project\113_Blank3.raw

------------------------------------------------------------------

Processing node 1: Select Spectra

------------------------------------------------------------------

1. General Settings:

- Precursor Selection: Use MS(n - 1) Precursor

- Use Isotope Pattern in Precursor Reevaluation: True

- Provide Profile Spectra: Automatic

- Store Chromatograms: False

2. Spectrum Properties Filter:

- Lower RT Limit: 1

- Upper RT Limit: 24

- First Scan: 0

- Last Scan: 0

- Ignore Specified Scans: (not specified)

- Lowest Charge State: 0

- Highest Charge State: 0

- Min. Precursor Mass: 70 Da

- Max. Precursor Mass: 5000 Da

- Total Intensity Threshold: 0

- Minimum Peak Count: 1

3. Scan Event Filters:

- Mass Analyzer: (not specified)

- MS Order: Any

- Activation Type: (not specified)

- Min. Collision Energy: 0

- Max. Collision Energy: 1000

- Scan Type: Any

- Polarity Mode: (not specified)

4. Peak Filters:

- S/N Threshold (FT-only): 0

5. Replacements for Unrecognized Properties:

- Unrecognized Charge Replacements: 1

- Unrecognized Mass Analyzer Replacements: ITMS

- Unrecognized MS Order Replacements: MS2

- Unrecognized Activation Type Replacements: CID

- Unrecognized Polarity Replacements: +

- Unrecognized MS Resolution@200 Replacements: 60000

- Unrecognized MSn Resolution@200 Replacements: 30000

------------------------------------------------------------------

Processing node 2: Align Retention Times

------------------------------------------------------------------

1. General Settings:

- Alignment Model: Adaptive curve

- Alignment Fallback: Use Linear Model

- Maximum Shift [min]: 0.7

- Shift Reference File: True

- Mass Tolerance: 5 ppm

- Remove Outlier: True

------------------------------------------------------------------

Processing node 3: Detect Compounds

------------------------------------------------------------------

1. General Settings:

- Mass Tolerance [ppm]: 5 ppm

- Intensity Tolerance [%]: 30

- S/N Threshold: 1.5

- Min. Peak Intensity: 10000

- Ions:

[2M+ACN+H]+1

[2M+ACN+Na]+1

[2M+H]+1

[2M+K]+1

[2M+Na]+1

[2M+NH4]+1

[M+ACN+H]+1

[M+ACN+Na]+1

[M+H]+1

[M+K]+1

[M+Na]+1

[M+NH4]+1

- Base Ions: [M+H]+1

- Min. Element Counts: C H

- Max. Element Counts: C150 H250 Br3 Cl4 K2 N10 Na2 O18 P5 S5

2. Peak Detection:

- Filter Peaks: True

- Max. Peak Width [min]: 0.5

- Remove Singlets: True

- Min. # Scans per Peak: 8

- Min. # Isotopes: 2

------------------------------------------------------------------

Processing node 4: Group Compounds

------------------------------------------------------------------

1. Compound Consolidation:

- Mass Tolerance: 5 ppm

- RT Tolerance [min]: 0.3

2. Fragment Data Selection:

- Preferred Ions: [M+H]+1

------------------------------------------------------------------

Processing node 16: Fill Gaps

------------------------------------------------------------------

1. General Settings:

- Mass Tolerance: 5 ppm

- S/N Threshold: 1.5

- Use Real Peak Detection: True

------------------------------------------------------------------

Processing node 19: Normalize Areas

------------------------------------------------------------------

1. QC-based Area Correction:

- Regression Model: Linear

- Min. QC Coverage [%]: 25

- Max. QC Area RSD [%]: 50

- Max. # Files Between QC Files: 20

2. Area Normalization:

- Normalization Type: None

- Exclude Blanks: True

------------------------------------------------------------------

Processing node 15: Mark Background Compounds

------------------------------------------------------------------

1. General Settings:

- Max. Sample/Blank: 5

- Max. Blank/Sample: 0

- Hide Background: True

------------------------------------------------------------------

Processing node 8: Search ChemSpider

------------------------------------------------------------------

1. Search Settings:

- Database(s): Human Metabolome Database; KEGG; LipidMAPS; PubMed

- Search Mode: By Formula or Mass

- Mass Tolerance: 5 ppm

- Max. # of results per compound: 100

- Max. # of Predicted Compositions to be searched per Compound: 3

- Result Order (for Max. # of results per compound): Order By Reference Count (DESC)

2. Predicted Composition Annotation:

- Check All Predicted Compositions: False

------------------------------------------------------------------

Processing node 22: Apply mzLogic

------------------------------------------------------------------

1. Search Settings:

- FT Fragment Mass Tolerance: 10 ppm

- IT Fragment Mass Tolerance: 0.4 Da

- Max. # Compounds: 0

- Max. # mzCloud Similarity Results to consider per Compound: 10

- Match Factor Threshold: 30

------------------------------------------------------------------

Processing node 9: Assign Compound Annotations

------------------------------------------------------------------

1. General Settings:

- Mass Tolerance: 5 ppm

2. Data Sources:

- Data Source #1: Predicted Compositions

- Data Source #2: ChemSpider Search

- Data Source #3: mzCloud Search

- Data Source #4: Metabolika Search

- Data Source #5: (not specified)

------------------------------------------------------------------

Processing node 12: Predict Compositions

------------------------------------------------------------------

1. Prediction Settings:

- Mass Tolerance: 5 ppm

- Min. Element Counts: C H

- Max. Element Counts: C250 H190 N10 O18 P5 S5

- Min. RDBE: 0

- Max. RDBE: 40

- Min. H/C: 0.1

- Max. H/C: 3.5

- Max. # Candidates: 10

- Max. # Internal Candidates: 200

2. Pattern Matching:

- Intensity Tolerance [%]: 30

- Intensity Threshold [%]: 0.1

- S/N Threshold: 3

- Min. Spectral Fit [%]: 30

- Min. Pattern Cov. [%]: 90

- Use Dynamic Recalibration: True

3. Fragments Matching:

- Use Fragments Matching: True

- Mass Tolerance: 10 ppm

- S/N Threshold: 3

------------------------------------------------------------------

Processing node 23: Map to KEGG Pathways

------------------------------------------------------------------

1. Search Settings:

- Search Mode: By Formula or Mass

2. By Mass Search Settings:

- Mass Tolerance: 5 ppm

3. By Formula Search Settings:

- Max. # of Predicted Compositions to be searched per Compound: 3

4. Display Settings:

- Max. # Pathways in 'Pathways' column: 20

------------------------------------------------------------------

Processing node 25: Map to Metabolika Pathways

------------------------------------------------------------------

1. Search Settings:

- Metabolika Pathways:

- Search Mode: By Formula or Mass

2. By Mass Search Settings:

- Mass Tolerance: 5 ppm

3. By Formula Search Settings:

- Max. # of Predicted Compositions to be searched per Compound: 3

4. Display Settings:

- Max. # Pathways in 'Pathways' column: 20

------------------------------------------------------------------

Processing node 21: Search mzCloud

------------------------------------------------------------------

1. Search Settings:

- Compound Classes: All

- Match Ion Activation Type: True

- Match Ion Activation Energy: Match with Tolerance

- Ion Activation Energy Tolerance: 20

- Apply Intensity Threshold: True

- Precursor Mass Tolerance: 10 ppm

- FT Fragment Mass Tolerance: 10 ppm

- IT Fragment Mass Tolerance: 0.4 Da

- Identity Search: HighChem HighRes

- Similarity Search: Similarity Reverse

- Library: Reference

- Post Processing: Recalibrated

- Match Factor Threshold: 60

- Max. # Results: 10

------------------------------------------------------------------

Processing node 26: Compound Class Scoring

------------------------------------------------------------------

1. General Settings:

- Compound Classes: \Carnitines

- S/N Threshold: 50

- High Acc. Mass Tolerance: 2.5 mmu

- Low Acc. Mass Tolerance: 0.5 Da

- Allow AIF Scoring: True

------------------------------------------------------------------

Processing node 10: Differential Analysis

------------------------------------------------------------------

1. General Settings:

- Log10 Transform Values: True

------------------------------------------------------------------

Processing node 18: Descriptive Statistics

------------------------------------------------------------------

No parameters

------------------------------------------------------------------

FILTERS USED:

- Background is false

- Area (Max.) is greater than 1000

- Formula is not blank

- # Usable QC is greater than 15

- Group CV [%] is less than 200 in every sample group

- RT [min] is less than 12

**References**

1. Kouřil Š, de Sousa J, Václavík J, Friedecký D, Adam T. CROP: Correlation-based reduction of feature multiplicities in untargeted metabolomic data. *Bioinformatics*. 2020:1(2). doi:10.1093/bioinformatics/btaa012

2. Dunn WB, Broadhurst D, Begley P, et al. Procedures for large-scale metabolic profiling of serum and plasma using gas chromatography and liquid chromatography coupled to mass spectrometry. *Nat Protoc*. 2011;6(7):1060-1083. doi:10.1038/nprot.2011.335

3. Gardlo A, Friedecký D, Najdekr L, Karlíková R, Adam T. Metabol: The statistical analysis of metabolomic data. 2019. doi:10.5281/zenodo.3235775

4. Dieterle F, Ross A, Schlotterbeck G, Senn H. Probabilistic quotient normalization as robust method to account for dilution of complex biuological mixtures. Application in 1H NMR Metabonomics. *Anal chem*. 2006;78(13):4281-4290.

5. Di Guida R, Engel J, Allwood JW, et al. Non-targeted UHPLC-MS metabolomic data processing methods: a comparative investigation of normalisation, missing value imputation, transformation and scaling. *Metabolomics*. 2016;12(5). doi:10.1007/s11306-016-1030-9

6. Hubert M, Rousseeuwm PJ, Vanden Branden K. ROBPCA: a new approach to robust principal component analysis. *Technometrics*. 2005;47(1):64-79. doi:10.1198/004017004000000563

7. de Sousa J, Vencálek O, Hron K, Václavík J, Friedecký D, Adam T. Bayesian Multiple Hypotheses Testing in Compositional Analysis of Untargeted Metabolomic Data. *Anal Chim Acta*. 2020;1097:49-61. doi:10.1016/J.ACA.2019.11.006
