# Supplementary material for: A newborn screening approach to diagnose 3‐hydroxy‐3‐methylglutaryl‐CoA lyase deficiency
Source: JIMD Rep. 2020 Apr 14;54(1):79–86. doi: 10.1002/jmd2.12118 (PMC7358667; doi:10.1002/jmd2.12118)

# A newborn screening approach to diagnose 3-hydroxy-3-methylglutaryl CoA lyase deficiency

# Supplement materials S2

Figure 1 An overview of performed experiments and data analysis.


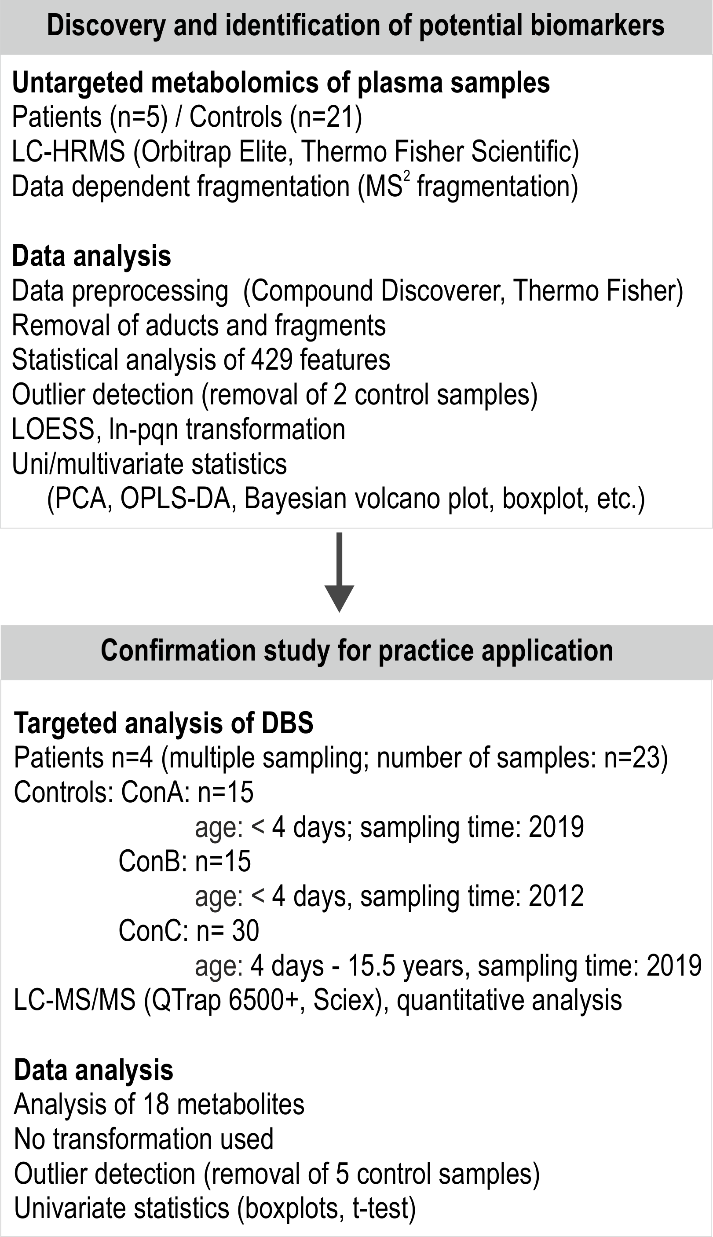

Supplement: Supplementary file 2 — Data S2. An overview of performed experiments and data analysis. [file JMD2-54-79-s002.docx]
