# Supplementary material for: A newborn screening approach to diagnose 3‐hydroxy‐3‐methylglutaryl‐CoA lyase deficiency
Source: JIMD Rep. 2020 Apr 14;54(1):79–86. doi: 10.1002/jmd2.12118 (PMC7358667; doi:10.1002/jmd2.12118)
Supplement: Supplementary file 3 — Data S3. LC‐MS/MS analysis of DBS. [file JMD2-54-79-s003.docx]

# A newborn screening approach to diagnose 3-hydroxy-3-methylglutaryl CoA lyase deficiency

# Supplement materials S3

### **Targeted LC-MS/MS analysis of DBS**

All samples were applied (4 µL) to the same column as in the case of untargeted metabolomic analysis and maintained at 30°C within the column compartment of LC system mentioned in Methods section. The buffers consisted of mobile phase A (0.5 % formic acid in water) and mobile phase B (0.5 % formic acid in ACN). At flow rate 350 µL/min, the analysis of one sample required 26 min. The gradient program was as follows: t = 0.0 – 2.0 min, 0 % B; t = 9.0 min, 20 % B; t = 17.0 – 20.0 min, 95 % B; 21.0 – 26.0 min, 0 % B. The temperature of the autosampler was held constant at 4 °C. Samples were analyzed in positive (+4.5 kV) and negative (-4.5 kV) mode using electrospray ionization. The temperature of the ion source was set to 450 °C. The curtain gas was set to 35 psi and both ion source gases were set to 50 psi.

Peak integration was done using MultiQuant 3.0 software (SCIEX) and evaluated in Microsoft Excel (MS Office 2016). Integrated peak areas of organic acids were divided by peak area of methylmalonate-D3 and all the other metabolites were divided by peak area of isovalerylcarnitine-D9. Concentrations of metabolites of interest were calculated based on a calibration curve. Based on ROBPCA outlier detection performed in the R package Metabol as described in Methods section, a total of 5 control samples was discarded (i.e., 1 ConA, 1 ConB, and 3 ConC).

Table 1. A complete list of monitored metabolites in targeted LC-MS/MS analysis in positive mode.

| Pos MRM | Q1 | Q3 | DP | CE |
| --- | --- | --- | --- | --- |
| C0 | 162.1 | 85.0 | 51 | 29 |
| C0-D9 | 171.1 | 85.1 | 51 | 29 |
| C5 | 246.2 | 85.1 | 56 | 29 |
| C5-D9 | 255.2 | 85.0 | 66 | 20 |
| 3MC-C | 244.1 | 84.9 | 41 | 29 |
| 3HIV-C | 262.2 | 85.0 | 60 | 30 |
| 3MGC-C | 288.2 | 85.0 | 41 | 29 |
| 3MG-C | 290.1 | 212.9 | 51 | 23 |
| C5-DC-D6 | 282.3 | 84.9 | 70 | 35 |
| 3H3MG-C | 306.2 | 85.0 | 51 | 23 |
| IV-Gly | 160.0 | 75.8 | 1 | 13 |
| 3MC-Gly | 158.0 | 82.8 | 6 | 15 |
| Leu | 132.0 | 85.9 | 43 | 17 |
| LeuLeu | 245.2 | 132.1 | 10 | 19 |
| inosine | 268.9 | 136.9 | 81 | 21 |
| mevalonolactone | 131.0 | 69,1 | 1 | 13 |

Table 2. A complete list of monitored metabolites in targeted LC-MS/MS analysis in negative mode.

| Neg MRM | Q1 | Q3 | DP | CE |
| --- | --- | --- | --- | --- |
| 3HIV-A | 116.9 | 58.9 | -25 | -14 |
| 3MGC-A | 142.9 | 98.9 | -5 | -10 |
| 3MG-A | 144.9 | 59.0 | -70 | -30 |
| 3H3MG-A | 160.9 | 98.9 | -25 | -16 |
| 2-OH isocaproate | 131.0 | 85.0 | -50 | -17 |
| adipate | 144.9 | 80.9 | -35 | -26 |
| acetoacetate | 101.0 | 57.2 | -50 | -15 |
| methylmalonate-D3 | 120.0 | 76.0 | -25 | -14 |
| hexanoyl-Gly ^13^C2 ^15^N | 175.0 | 77.0 | -10 | -16 |

**Setting of MS instrument in targeted LC-MS/MS analysis:**

- Potential: +4500/-4500 V
- Curtain Gas: 35 AU
- Temperature: 450 °C
- Ion Source Gas 1/Gas 2: 50/50 AU
- Target Cycle Time: 0.9 s
- MRM window: 60 s
- Settling: 10 ms
- Pause time: 5 ms

**Abbreviations**

3H3MG-A        3-hydroxy-3-methylglutaric acid

3H3MG-C        3-hydroxy-3-methylglutarylcarnitine

3HIV-A            3-hydroxyisovaleric acid

3HIV-C            3-hydroxyisovalerylcarnitine

3MC-C            3-methylcrotonylcarnitine

3MC-Gly 3-methylcrotonylglycine

3MG-A            3-methylglutaric acid

3MG-C            3-methylglutarylcarnitine

3MGC-A        3-methylglutaconic acid

3MGC-C        3-methylglutaconylcarnitine

C0 carnitine

C0-D9 carnitine-D9

C5 C5-carnitine

C5-D9 C5-carnitine-D9

C5-DC-D9 C5-dicarboxylic carnitine-D9

IV-C isovalerylcarnitine

IV-Gly isovalerylglycine
