# Supplementary material for: A newborn screening approach to diagnose 3‐hydroxy‐3‐methylglutaryl‐CoA lyase deficiency
Source: JIMD Rep. 2020 Apr 14;54(1):79–86. doi: 10.1002/jmd2.12118 (PMC7358667; doi:10.1002/jmd2.12118)
Supplement: Supplementary file 4 — Data S4. Two‐dimensional score plot of unsupervised PCA analysis of HMGCLD patient plasma (Pt, blue) and controls (Con, pink). The tight green cluster of QC samples show a good stability of the analysis. Circled areas represent 75% confidence ellipses. [file JMD2-54-79-s004.docx]

# A novel screening approach to diagnose 3-hydroxy-3-methylglutaryl-CoA lyase deficiency

# Supplement materials S4


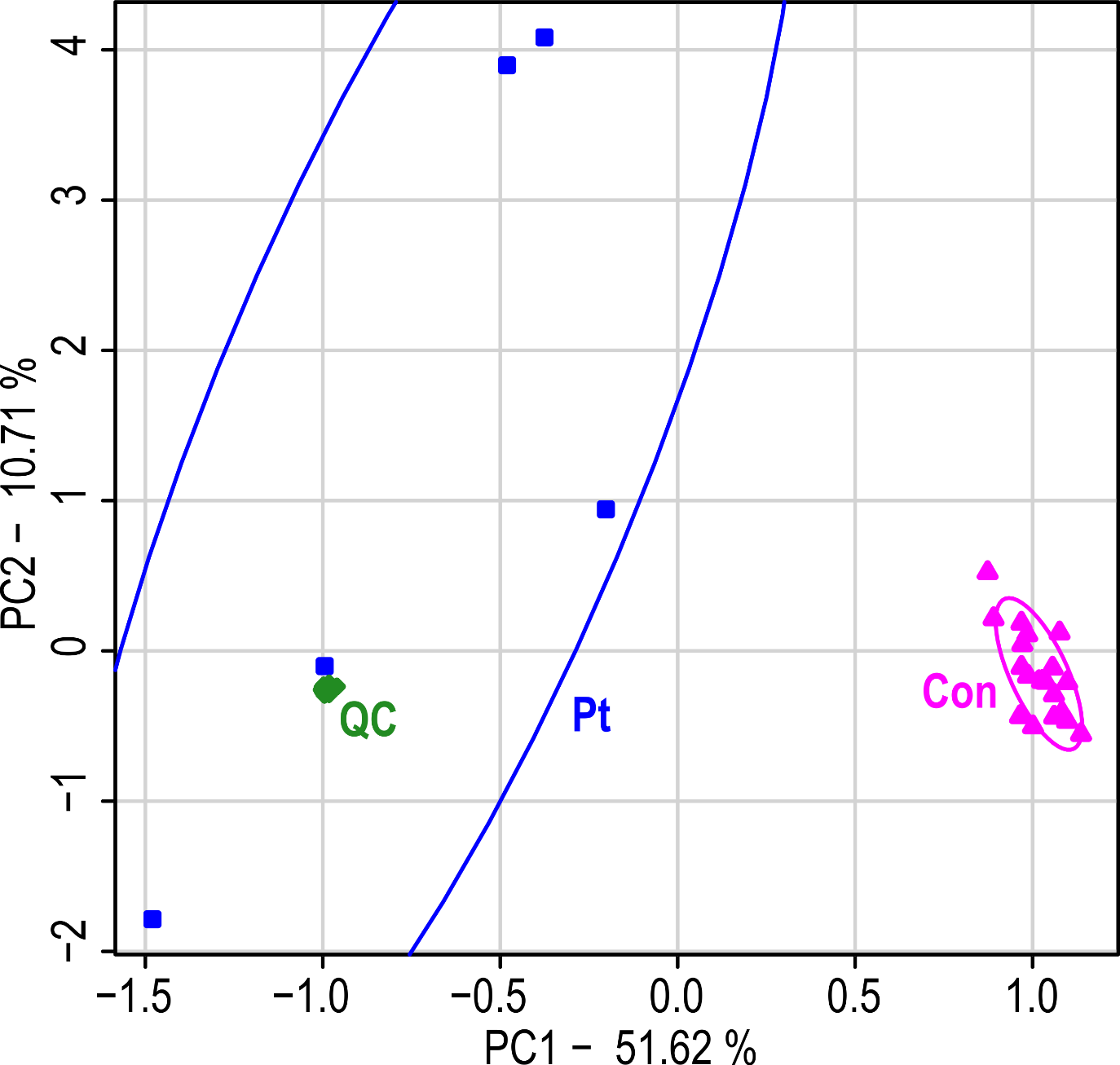


*Figure 1. Two-dimensional score plot of unsupervised PCA analysis of HMGCLD patient plasma (Pt, blue) and controls (Con, pink). The tight green cluster of QC samples show a good stability of the analysis. Circled areas represent 75% confidence ellipses.*
