# Supplementary material for: A newborn screening approach to diagnose 3‐hydroxy‐3‐methylglutaryl‐CoA lyase deficiency
Source: JIMD Rep. 2020 Apr 14;54(1):79–86. doi: 10.1002/jmd2.12118 (PMC7358667; doi:10.1002/jmd2.12118)
Supplement: Supplementary file 5 — Data S5. Bayesian volcano plot with colored HDI distance levels for the untargeted analysis. The dark red points stand for the non‐significant compounds. The potential biomarkers are depicted in blue‐green colors, located in the upper right corner for patients and in the upper left corner for controls, respectively. *No metabolites detected in these HDI distance levels. [file JMD2-54-79-s005.docx]

# A novel screening approach to diagnose 3-hydroxy-3-methylglutaryl-CoA lyase deficiency

# Supplement materials S5


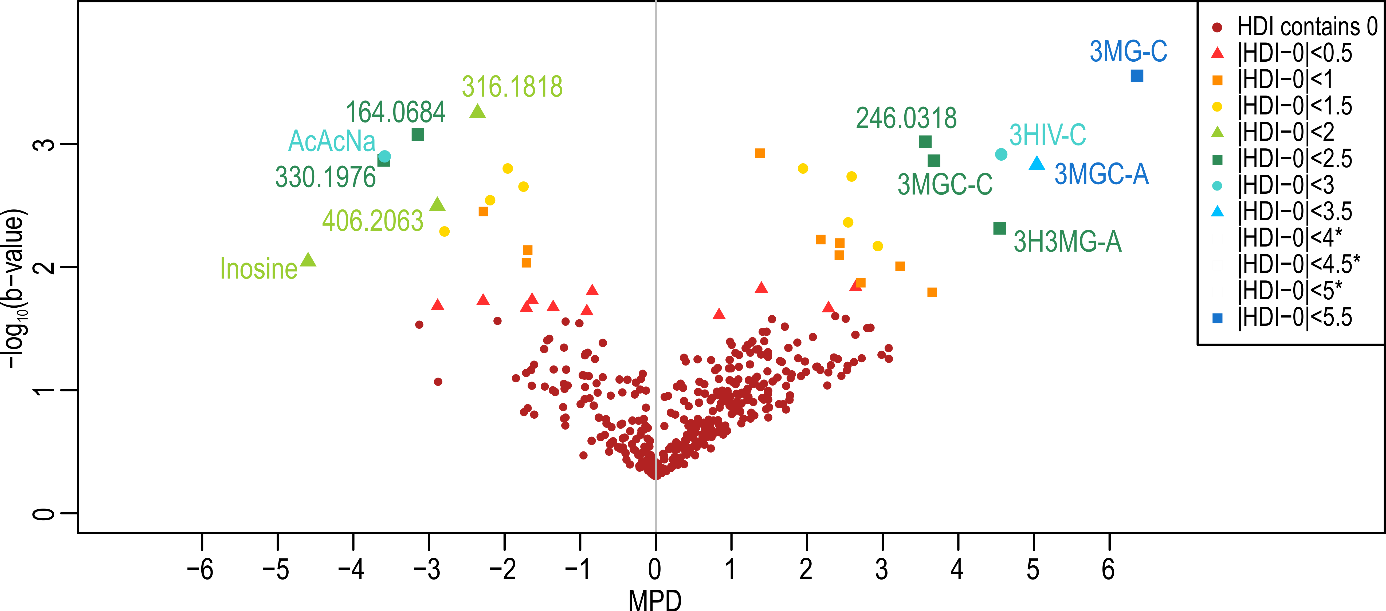


*Figure 1. Bayesian volcano plot with colored HDI distance levels for the untargeted analysis. The dark red points stand for the non-significant compounds. The potential biomarkers are depicted in blue-green colors, located in the upper right corner for patients and in the upper left corner for controls, respectively.
* No metabolites detected in these HDI distance levels.*
