# Supplementary material for: A newborn screening approach to diagnose 3‐hydroxy‐3‐methylglutaryl‐CoA lyase deficiency
Source: JIMD Rep. 2020 Apr 14;54(1):79–86. doi: 10.1002/jmd2.12118 (PMC7358667; doi:10.1002/jmd2.12118)
Supplement: Supplementary file 6 — Data S6. Acylcarnitine profile of five HMGCLD patients compared to 19 healthy controls in plasma. Elevated organic acids found in plasma samples of HMGCLD patients compared to 19 healthy controls. [file JMD2-54-79-s006.docx]

# A novel screening approach to diagnose 3-hydroxy-3-methylglutaryl-CoA lyase deficiency

# Supplement materials S6

Apart from known plasmatic biomarkers of HMGCLD patients (3HIV-C, 3MG-C and 3MGC-C) three other acylcarnitine species and free organic acids derived from intermediates in the leucine degradation pathway were observed elevated using untargeted metabolomic analysis, see Table 1 and 2.

Table 1. Acylcarnitine profile of five HMGCLD patients compared to 19 healthy controls in plasma.

| acylcarnitine | mean peak area of 19 controls | 2SD | mean peak area of 5 patients | 2SD | fold-change |
| --- | --- | --- | --- | --- | --- |
| IV-C | 2712 | 3143 | 6761 | 12550 | 2.5 |
| 3MC-C | 35 | 18 | 716 | 1742 | 20.4 |
| 3HIV-C | 619 | 899 | 84847 | 198406 | 137.1 |
| 3MGC-C | 19 | 243 | 952 | 1411 | 50.5 |
| 3MG-C | 108 | 199 | 6797 | 44782 | 63.2 |
| 3H3MG-C | 14 | 10 | 121 | 275 | 8.4 |

Table 2. Elevated organic acids found in plasma samples of HMGCLD patients compared to 19 healthy controls.

| organic acid | mean area of 19 controls | 2SD | mean area of 5 patients | 2SD | fold-change |
| --- | --- | --- | --- | --- | --- |
| 3HIV-A | 2141 | 2068 | 148054 | 530638 | 69.2 |
| 3MGC-A | 419 | 286 | 63345 | 87226 | 151.4 |
| 3H3MG-A | 413 | 398 | 148429 | 529361 | 359.4 |
